# Management of Post-tubercular Hemoptysis With Bronchial Artery Embolization: A Case Report

**DOI:** 10.7759/cureus.98626

**Published:** 2025-12-07

**Authors:** Jyoti Bajpai, Shubhajeet Roy, Ankit Sharma, Saurabh Kumar, Surya Kant

**Affiliations:** 1 Respiratory Medicine, King George's Medical University, Lucknow, IND; 2 Anesthesiology and Critical Care, King George's Medical University, Lucknow, IND; 3 Radiodiagnosis, King George's Medical University, Lucknow, IND

**Keywords:** bronchial artery embolization, computed tomography angiography, intervention radiology, massive hemoptysis, tuberculosis

## Abstract

Pulmonary tuberculosis is a known cause of massive hemoptysis, which can be a life-threatening complication. This case explores the complexities of recurrent hemoptysis in a patient with a history of treated pulmonary tuberculosis. A 55-year-old gentleman with a history of pulmonary tuberculosis presented with progressive breathlessness and copious hemoptysis. Imaging revealed bronchiectasis, and computed tomography angiography indicated dilated bronchial arteries. Despite symptomatic management, the patient's condition deteriorated, necessitating multidisciplinary interventions. Bronchial artery embolization (BAE) successfully resolved the symptoms. Massive hemoptysis in pulmonary tuberculosis, though uncommon, demands thorough evaluation. Selective BAE has emerged as a crucial intervention. This case highlights the importance of a collaborative approach involving respiratory medicine and interventional radiology in managing complex cases of massive hemoptysis. BAE stands out as a viable and successful intervention in such scenarios.

## Introduction

The expectoration of blood from the respiratory system is known as hemoptysis. Lung cancer, chronic bronchitis, and bronchiectasis (permanent dilatation of the airways) are the most common causes. The bronchial arteries (which supply oxygenated blood to the lung parenchyma itself, as opposed to pulmonary circulation, which carries deoxygenated blood for gas exchange with the alveoli) are typically the source of expectorated blood [1]. Blood-streaked sputum, severe hemoptysis (frank blood), and major hemoptysis (huge volumes of fresh blood >100mL) are the three severity levels of hemoptysis [1]. Hemoptysis in pulmonary tuberculosis is frequently observed in cases of bronchial tuberculosis. It is caused by inflammation of the airways, parenchymal damage, rupture of pulmonary capillaries, or rupture of Rasmussen aneurysm (a pseudoaneurysm in which there is damage to the arterial wall, in the vicinity of a tuberculous cavity) in the pulmonary artery [2]. By describing the associated symptoms, the blood's appearance, and the patient's concurrent comorbidities, upper respiratory tract bleeding must be differentiated from hematemesis. This may be facilitated by endoscopic procedures such as bronchoscopy, gastroscopy, and/or rhinolaryngoscopy [3]. To determine the severity, bleeding must also be measured [3]. Up to 5-10% of individuals with previous pulmonary tuberculosis develop significant post-tubercular hemoptysis, which entails a substantial risk of asphyxiation and death if left untreated [4]. The diagnostic and therapeutic challenges of recurrent severe hemoptysis in a post-tubercular patient are showcased in this case report, with a highlight on the function of bronchial artery embolization (BAE) as a minimally invasive and effective life-saving intervention when medical management proves to be insufficient. The article highlights the importance of multidisciplinary teamwork in these scenarios by outlining the clinical presentation, imaging results, interventional strategy, and patient outcome.

## Case presentation

A 55-year-old man came to the respiratory medicine department's emergency room complaining of subtle, progressive dyspnea upon exertion that had progressed from modified Medical Research Council (mMRC) grade I to III over the course of a year. Along with a year of expectoration and yellowish, blood-stained, copious sputum, he also reported intermittent hemoptysis for a month (approximately 100 mL per day, two to three times per day; the last episode of hemoptysis occurred on the day of presentation: 150 mL of frank red blood with some clots). In addition, he had a history of pulmonary tuberculosis. Twelve years ago, he received anti-tubercular therapy (ATT) for six months after testing positive for acid-fast bacilli (AFB) in his sputum. His symptoms then improved. Three months ago, an AFB-positive sputum smear resulted in another diagnosis of pulmonary tuberculosis, and he is currently on an ATT. He has never seen seasonal variations in his symptoms or atopy. The patient did not have a history of type II diabetes mellitus or hypertension. He smoked about one bundle of beedi every day for 30 years. He also had a history of substance misuse, including using ganja, charas, hashish, and other drugs for the same amount of time. A pulmonary function test revealed that he did not have chronic obstructive pulmonary disease (COPD) (forced expiratory volume in one second to forced vital capacity ratio (FEV_1_/FVC) > 70%).

On physical examination, he had pallor and had shortness of breath with rapid, shallow breathing efforts. Cyanosis or clubbing was absent. Jugular venous pressure was within normal ranges, and there were no signs of pedal edema. Chest movements were bilaterally restricted. Trachea was central, and tactile vocal fremitus was focally less resonant at places. A dull percussion note was heard on the right side. There were coarse crackles heard on the right side, with bronchial breath sounds in some areas. His vital signs at admission indicated hypotension (blood pressure: 101/62 mmHg), tachycardia (heart rate: 102 beats per minute), and low oxygen saturation (SpO_2_: 82-84% on room air). Oxygen and fluid assistance were administered. The patient was put on vasopressor support since the blood pressure did not improve even after a 30 mL/kg fluid infusion. Broad-spectrum antibiotics were administered coupled with an infusion of paracetamol, and a blood culture was sent. The electrocardiogram suggested sinus tachycardia. Because the arterial blood gas measurement indicated type 2 respiratory failure, doxofylline infusion and oxygen titration were initiated in order to keep SpO_2_ between 88% and 92%. Nebulization was avoided since the patient had active hemoptysis.

The results of his serial investigation are reported in Table 1. Hemoglobin was less than the normal range, while the white cell count was within normal ranges. Initially, neutrophilia was detected. Prothrombin time was slightly raised. There were no signs of dyselectrolytemia (except hypocalcemia) or liver or kidney failure.

**Table 1:** Values of various laboratory parameters of the patient. Deranged values are shown in bold.

Parameter	Day 1	Day 4	Day 8	Day 11	Reference Range
Complete Blood Count					
Hemoglobin (g/dL)	8.9	11	12.9	13.2	13.8-17.2
Total Leukocyte Count (/mcL)	8300	7000	4800	5300	4000-11000
Differential Leukocyte Count					
Neutrophil (%)	80	70	72	63	40-60
Lymphocytes (%)	15	24	24	30	20-40
Platelet Count (x10^5^/mcL)	0.96	1.6	1.65	1.73	1.5-4.5
Electrolytes					
Serum Sodium (Na+) (mEq/L)	135	133	135	138	135-145
Serum Potassium (K+) (mmol/L)	4.9	3.4	4.3	4.4	3.6-5.2
Serum Calcium (Ca2+) (mg/dL)	4.8	4.4	4.0	4.1	8.5-10.2
Kidney Function Test					
Serum Protein (g/dL)	6.6	7.2	7.2	7.6	6.0-8.3
Serum Albumin (g/dL)	3.5	4.0	3.8	4.2	3.4-5.4
Serum Blood Urea Nitrogen (mg/dL)	27	28	20	18	7-30
Serum Creatinine (mg/dL)	0.82	0.68	0.65	0.73	0.74-1.35
Liver Function Test					
Serum Total Bilirubin (mg/dL)	0.3	0.4	0.45	0.5	0.1-1.2
Serum Glutamic Oxaloacetic Transaminase (U/L)	56	30	21	41	8-45
Serum Glutamic Pyruvic Transaminase (U/L)	34	22	21	29	7-56
Serum Alkaline Phosphatase (U/L)	152	141	123	128	44-147
Coagulation Profile					
Prothrombin Time (s)	16	-	14	-	11-13.5
International Normalized Ratio	1.3	-	1.1	-	<=1.1
HbA1c (%)	5.5	-	-	-	<5.7

**Figure 1:**
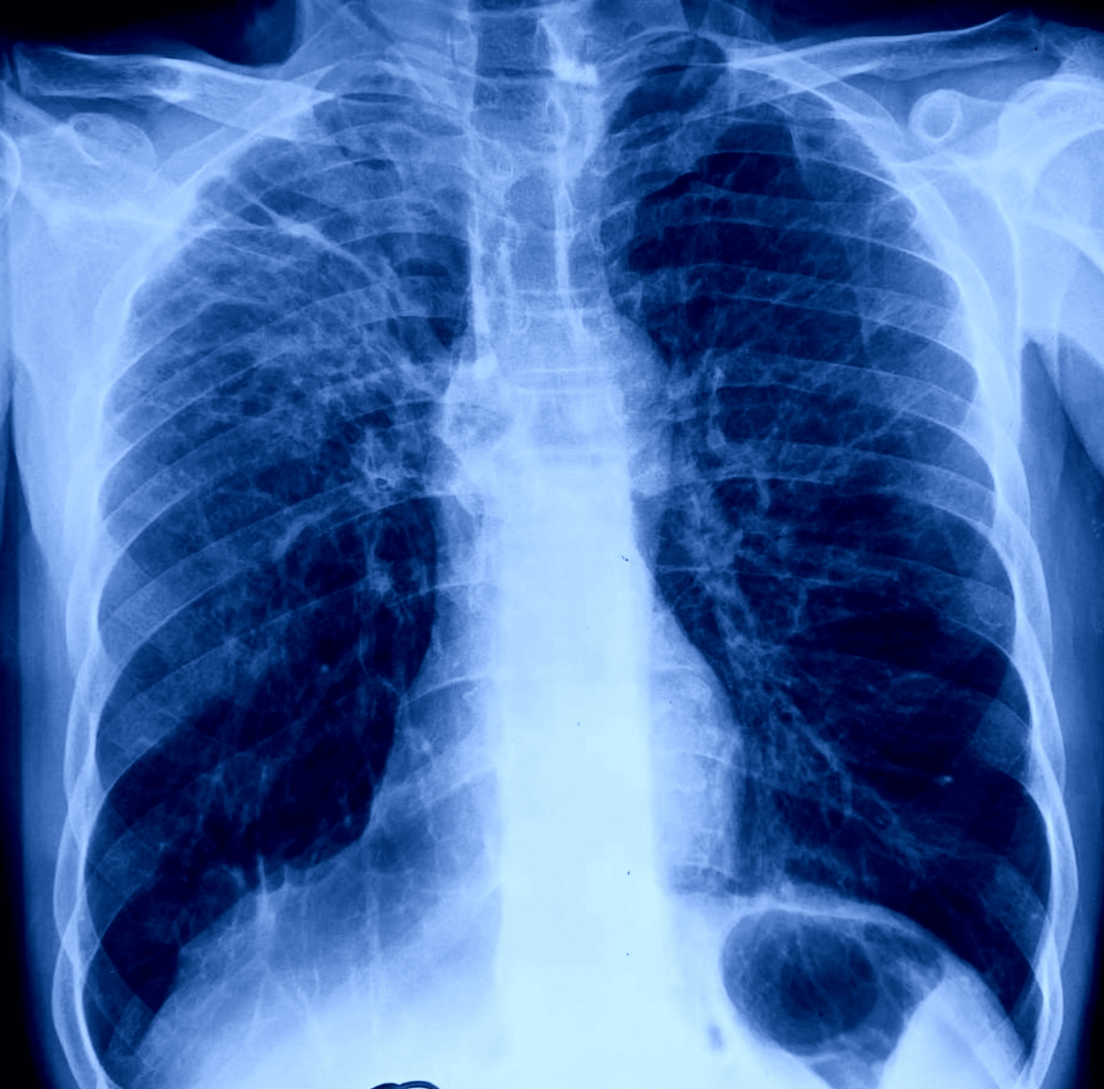
Posteroanterior chest X-ray suggestive of pulmonary tuberculosis.

The patient was found to be non-reactive for HIV. Pulmonary tuberculosis was suggested by the chest X-ray (posterio-anterior aspect) (Figure 1).

**Figure 2:**
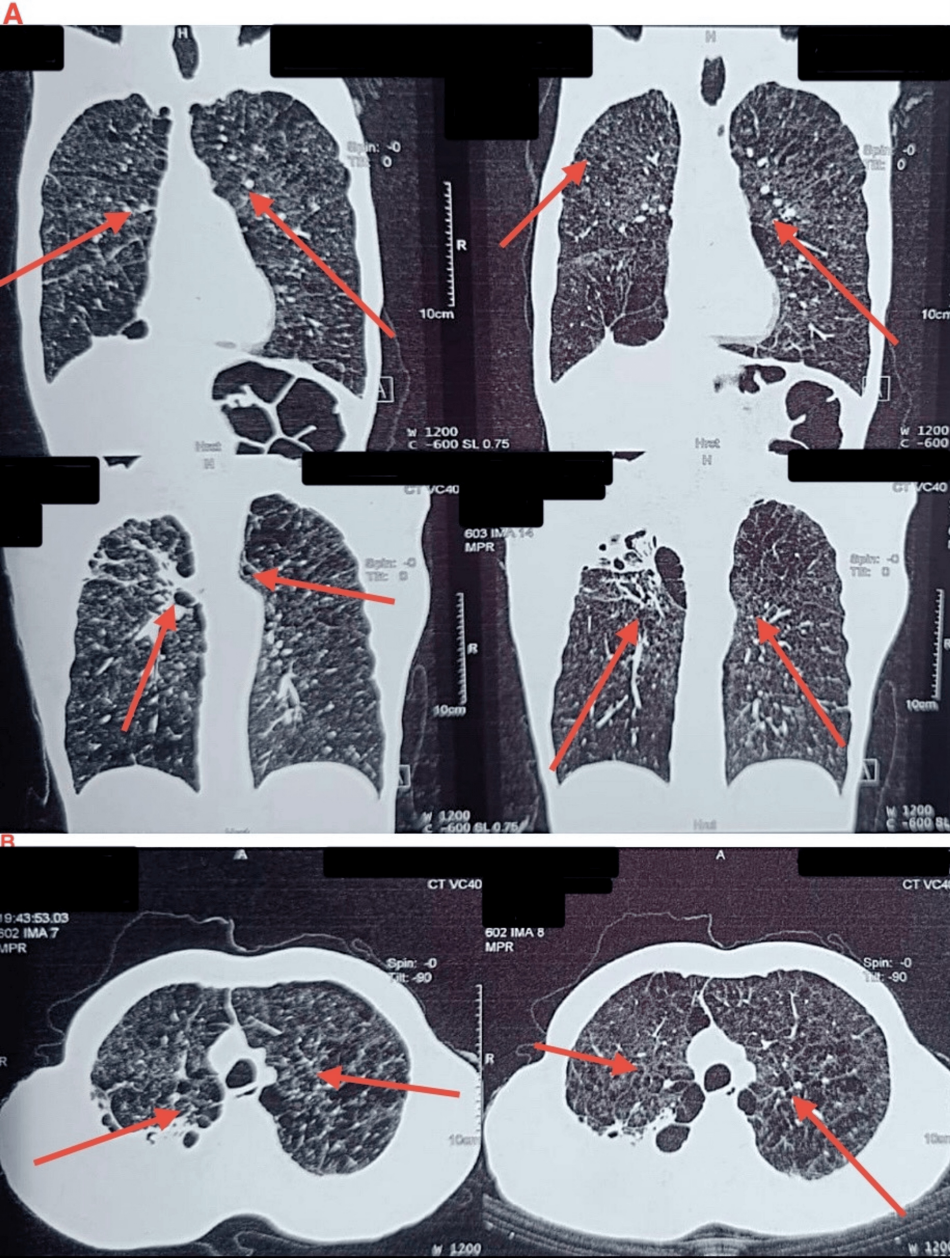
Contrast-enhanced CT of the thorax: (a) coronal section and (b) axial section, suggestive of bronchiectasis (red arrows).

A thoracic contrast-enhanced computed tomography (CECT) scan revealed bronchiectasis (Figure 2).

**Figure 3:**
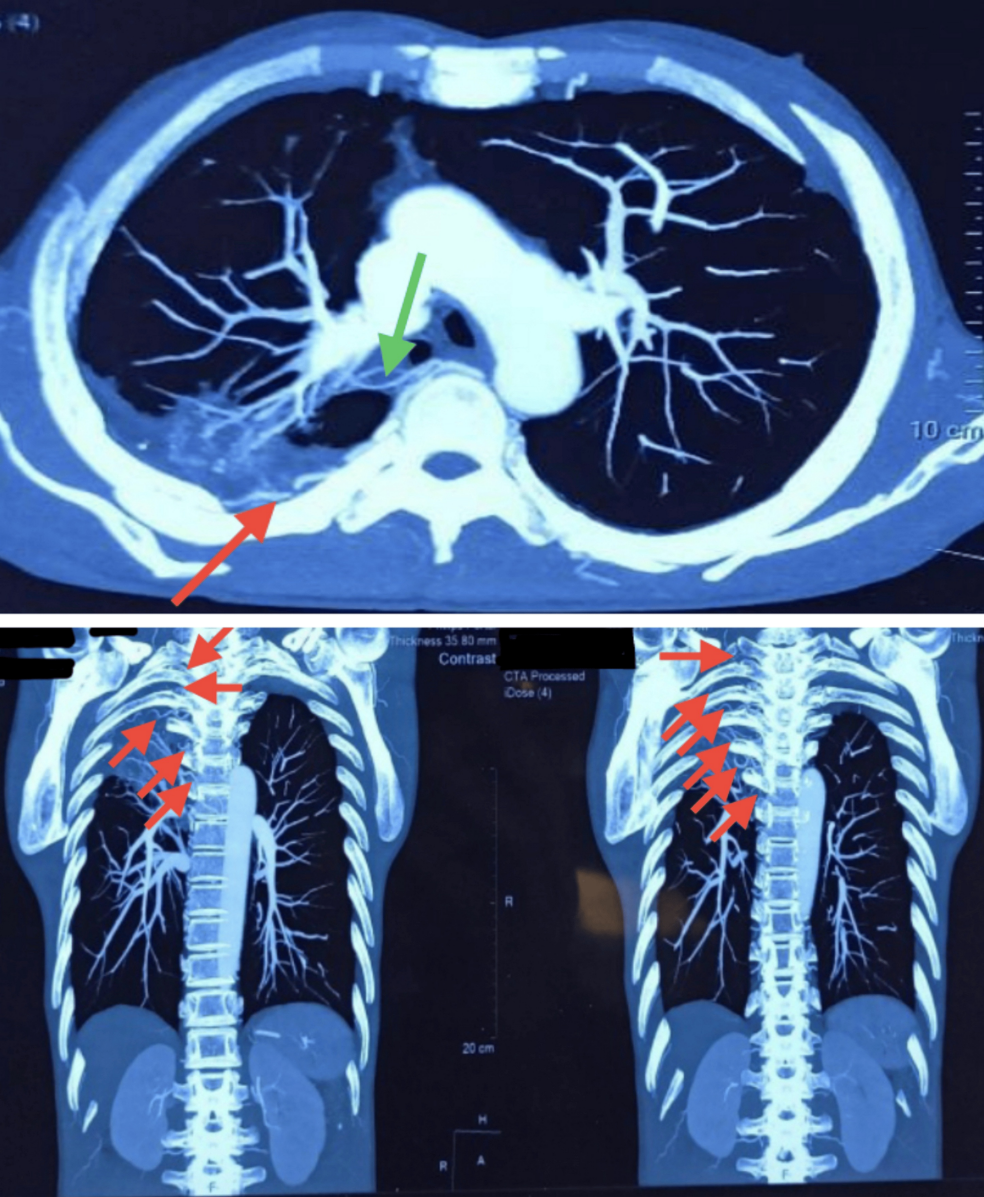
CT angiography of the thorax showing a dilated and tortuous right bronchial artery (green arrow) and right first to sixth intercostal arteries (red arrows).

A CT angiography revealed that the right bronchial artery was tortuous and dilated. Additionally, Figure 3 shows that the right intercostal arteries from the first to the sixth were tortuous and dilated.

**Figure 4:**
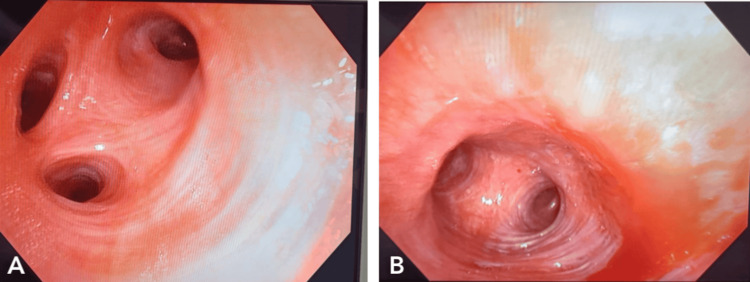
(A-B) Bronchoscopy showing no active bleeding site, patent segmental bronchi of the left and right tracheobronchial tree, and healthy mucosa.

For hemoptysis, a bronchoscopy was also scheduled. The segmental bronchi of the left and right tracheobronchial tree were patent, the mucosa was healthy, and bronchoscopy revealed no active bleeding site (Figure 4).

**Figure 5:**
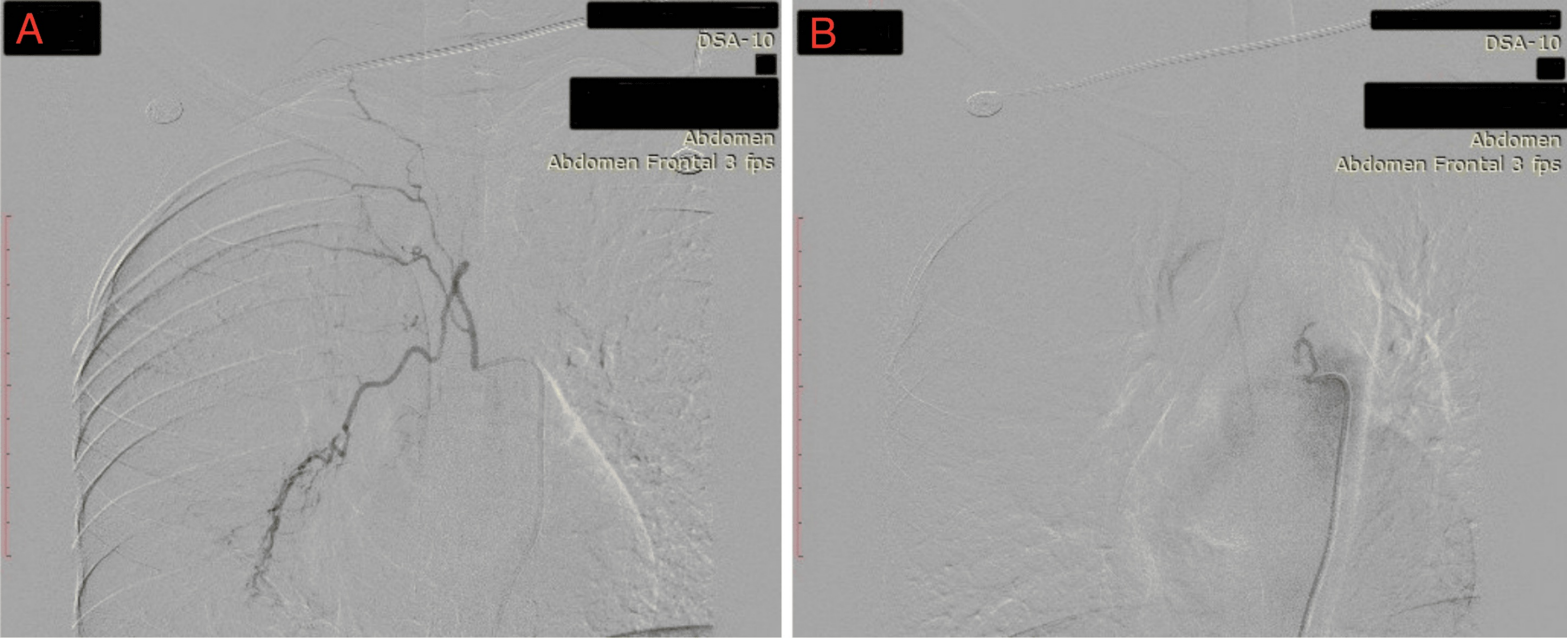
Bronchial artery embolization: (A) pre-procedure digital subtraction angiography and (b) post-procedure digital subtraction angiography.

BAE was suggested based on the CT angiography results, according to the intervention radiology department's report. As a result, the patient was placed in day care and moved to interventional radiology. Under general anesthesia, a 5-Fr vascular sheath was introduced via the right femoral artery, and vascular access was secured. The right bronchial artery, the right third and fourth intercostal arteries, and the right lateral thoracic artery all displayed aberrant blush (abnormal parenchymal blush: hypertrophied and tortuous), according to the selective bronchial and intercostal arteriography. Before embolization was done, systemic-pulmonary anastomoses and spinal artery feeders (especially the artery of Adamkiewicz) were meticulously screened for and ruled out to prevent any non-target embolization. Coil embolization was performed using an 18.3 mm detachable coil in the right thoracic artery (one coil), right bronchial (two coils), and the third intercostal (one coil). A 300x500 mcm particle of polyvinyl alcohol (PVA) was injected into the right lateral thoracic artery until near-stasis could be achieved. An angiography obtained after the treatment revealed no unusual flush and no signs of non-target embolization. The intervention was tolerated well by the patient, with no signs of any complication (Figure 5).

Rationale

BAE is the minimally invasive intervention of choice for severe and fatal hemoptysis, especially when medical management does not suffice, or when the bleeders are bronchial or systemic in origin. In our case, we embolized the right bronchial, third and fourth intercostal, and right lateral thoracic arteries, as per the angiography reports, as mentioned above. These arteries generally form a collateral supply of bronchiectatic regions following tuberculosis cavitation. The duo of coil-based mechanical occlusion and distal penetration with PVA particles covers both proximal as well as distal vessel occlusion, significantly decreasing the probabilities of early rebleed. Pre-embolization non-target embolization screening is important to prevent unwanted occlusion of spinal or esophageal arteries, which might precipitate symptoms owing to ischemia [5,6].

**Figure 6:**
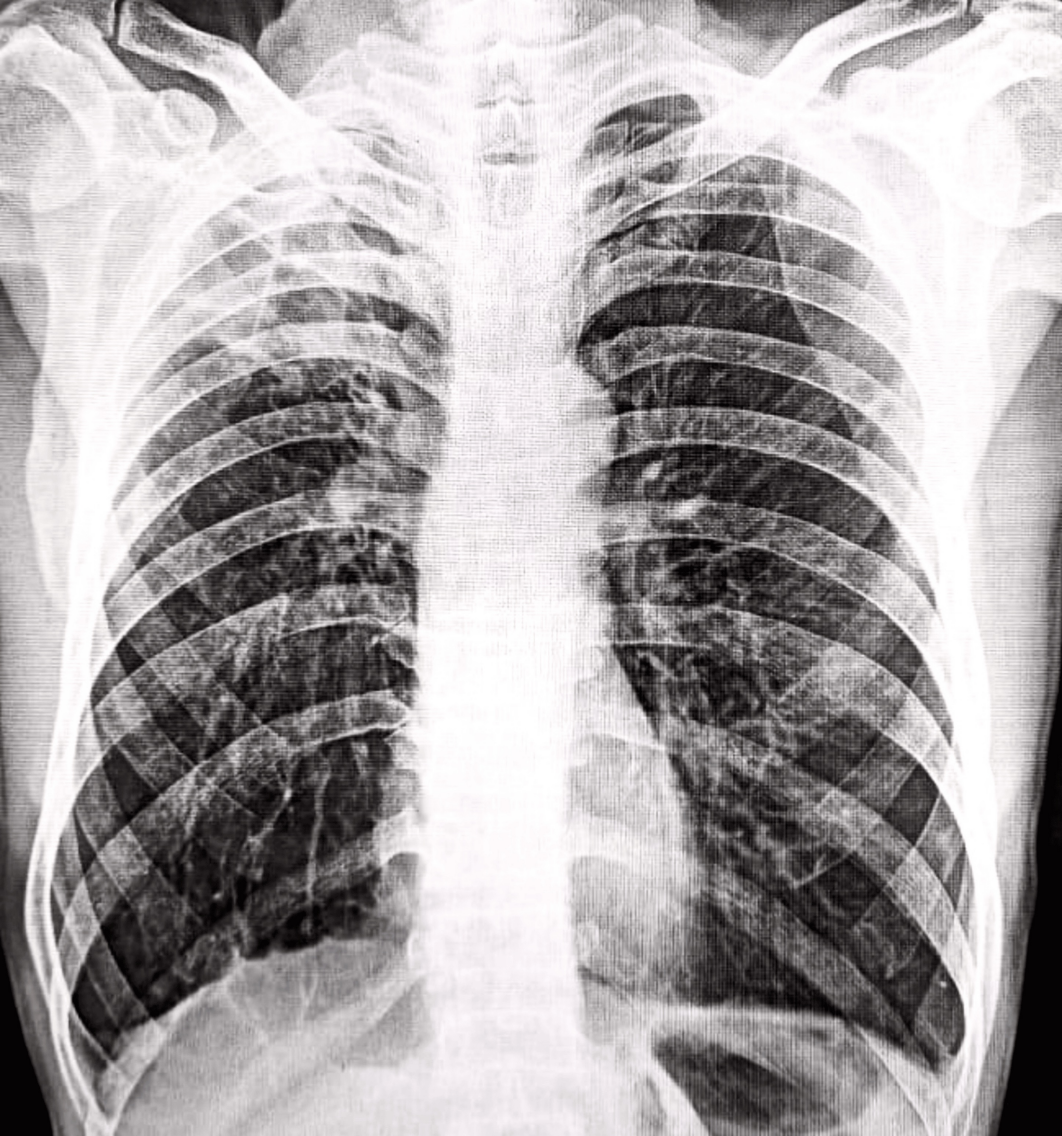
Post-procedure chest X-ray following bronchial artery embolization.

Figure 6 displays the X-ray taken after the procedure, which shows no new parenchymal opacities, pleural effusion, or pneumothorax, which in turn demonstrates radiographic stability post-intervention, and correlates with the patient’s subsequent cessation of symptoms.

After that, the patient's symptoms resolved, and he was doing well on routine follow-up till one year post-intervention, with no signs of recurrence, no indications for repeat BAE, hemoglobin values improved over time, and no complications were reported post-procedure. It was suggested that ATT be maintained.

## Discussion

Massive hemoptysis as a result of pulmonary tuberculosis is a lethal complication, occurring due to the erosive damage in the walls of the pulmonary vessels, bronchiectasis, and hypertrophy of systemic collaterals, including the bronchial and intercostal arteries. In spite of microbiological remission, these anatomical changes remain, and the patients become vulnerable to lethal rebleeding episodes [2,7]. According to the Global Tuberculosis Report 2019, 10 million cases were reported worldwide in 2018, 11% of which reported massive hemoptysis in their symptomatology [8]. India is one of the 30 countries with a high burden of tuberculosis, and the epidemic situation of massive hemoptysis in pulmonary tuberculosis is not optimistic. Hemoptysis occurs in patients with caseous, invasive pulmonary tuberculosis [2]. As in our case, massive hemoptysis happened in the patient, who had already been treated for pulmonary tuberculosis, and again got detected with pulmonary tuberculosis. Even after extensive symptomatic management, the patient could not be cured; hence, we headed for other investigations like CECT thorax, CT angiography, and bronchoscopy to rule out any other underlying disorder. On CT angiography, right bronchial artery dilatation was observed, and hence, the patient was managed by right BAE.

In chronic, devastating phenotypes of pulmonary TB, BAE single-handedly has proven to have an upper hand in conservative treatment, especially due to the fact that medical management is unable to undo the collateral vessel hypertrophy. Numerous studies have stressed the fact that timely embolization stabilizes acute bleeding as well as minimizes short-term recurrence in combination with adequate anti-tubercular medications and preventive therapies, which include minimization of risk factors, including but not exclusively smoking cessation [9]. BAE following post-tubercular hemoptysis is indicated in cases of hemoptysis resulting in significant airway compromise or respiratory distress, or three or more episodes of hemoptysis with ≥100 mL blood loss within one week, or chronic or slowly increasing bleeding episodes [10]. However, it is contraindicated in cases of coagulopathy, severe allergic reactions, chronic kidney disease, severely hypertensive patients, and in cases of pregnancy [10].

It is advisable to get a thoracic aortogram done before a selective BAE [11]. Selective catheterization can be done by various systems, like Cobra, Simmons, Shepherd's crook, Mikaelsson, or Yashiro catheters [11]. After selective catheterization of the bronchial artery, an angiogram is done [11]. Particles used for BAE include PVA particles (permanent embolic agent) and gelatin sponge (temporary embolic agent). Stainless steel materials are avoided. The favorable particle size for BAE is 250-500 μm [6,12]. Newer techniques using a dual approach with coils and particulate agents, as in the present case, have enhanced both immediate and mid-term outcomes by enabling improved targeting of both proximal and distal feeding vessels [5].

Even though unarguable that BAE is rated highly in terms of effectiveness, studies report recurrence rates in the range of 10-30%, most commonly because of inadequate embolization, recanalization, or evolution of novel systemic collaterals. Patients with already existing diffuse bronchiectasis or current cavitary diseases are predisposed to a greater degree of risk. Follow-up radiology, ideally by CTA, is quite essential and necessary in high-risk patients [13].

It is pertinent to have knowledge of the potential complications, which may include spinal artery embolization, esophageal ischemia, and chest pain; however, meticulous pre-embolization mapping and appropriate use of ideally sized PVA particles can dramatically reduce the incidence of such serious complications [6].

## Conclusions

Though hemoptysis is not a rare finding in patients visiting the respiratory medicine ward, massive hemoptysis is an alarming condition. The etiology can be manifold, and the situation can become complicated by other co-existing pathologies of the respiratory system. A multidisciplinary approach with professionals from both respiratory medicine and intervention radiology can help in better tackling the challenge. BAE has had a good track record in tackling this issue, as also supported by this case and prior reports.
